# Optic Nerve Avulsion and Retinal Detachment After Penetrating Ocular Trauma: Case Report

**DOI:** 10.4274/tjo.31549

**Published:** 2018-04-25

**Authors:** Mehmet Fatih Kağan Değirmenci, Nilüfer Yalçındağ, Hüban Atilla

**Affiliations:** 1 Ankara University Faculty of Medicine, Department of Ophthalmology, Ankara, Turkey

**Keywords:** Ocular trauma, optic nerve, avulsion, retinal detachment

## Abstract

Optic nerve avulsion is a rare pathology with poor prognosis usually seen after blunt trauma. The optic nerve is separated from the sclera by indirect forces due to the relatively weak structure of the lamina cribrosa area. Here we describe an 11-year-old boy who experienced optic nerve avulsion and retinal detachment after penetrating ocular trauma.

## Introduction

Ocular trauma is among the leading causes of vision loss in children. In a population-based study conducted in the United States of America, the annual incidence of ocular trauma in children was found to be 15.2/100,000.^1^ Studies conducted in other developed countries have yielded similar results, but the incidence is somewhat higher in developing countries. Traumatic optic neuropathy is seen in 0.5-5% of all head traumas.^2^

Avulsion of the optic nerve is a rare complication after ocular trauma but carries a poor prognosis. Traumatic optic nerve damage can occur via direct and indirect mechanisms in different parts of the optic nerve. These mechanisms can include anterior displacement of the globe, retraction of the nerve, sudden rotational movement of the globe, or sudden rise in intraocular pressure.^3^ Due to the absence of connective tissue between the optic nerve fibers in the lamina cribrosa region and the absence of myelin sheathing around the nerve fibers, the optic nerve head is a relatively weak structure.

## Case Report

**Figure 1:**
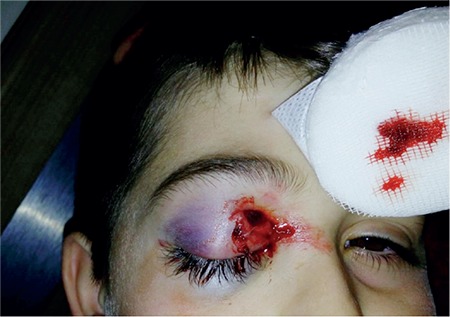
Ecchymosis of the upper and lower lids and laceration of the upper lid at presentation

An 11-year-old boy was brought to the pediatric emergency department due to a right eyelid injury sustained after falling from a tree. Systemic evaluation was normal and he was referred to the ophthalmology department. The patient reported having fallen onto a branch fragment from the tree approximately one hour earlier. Edema and ecchymosis of the right upper and lower lids, and a cutaneous wound in the nasal aspect of the right upper lid were observed on examination. Visual acuity was suspected light perception in the right eye and 10/10 in the left eye. Color vision and eye movements were normal in the patient’s left eye but could not be evaluated in his right. The right pupil was middilated with intact consensual but no direct light response. The left eye exhibited normal direct but absent consensual pupillary light reflexes. Anterior segment examination of the right eye revealed hyperemic conjunctiva, clear cornea, and +1 cells in the anterior chamber. The fundus could not be evaluated due to vitreous hemorrhage. Anterior and posterior examinations in the left eye were normal. The patient was admitted to our unit for wound exploration, repair of the lid wound, and fundus examination under general anesthesia (Figure 1). With a prediagnosis of traumatic optic neuropathy, treatment was initiated with systemic steroids, and topical steroids for the anterior chamber reaction, and bed rest in an upright position was recommended.

**Figure 2:**
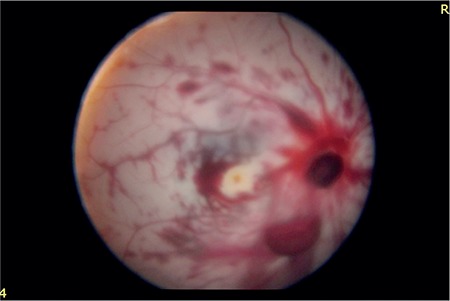
Fundus photograph in the right eye taken one day after the trauma

The following day, the patient underwent wound site exploration and primary incision repair under general anesthesia, followed by fundus examination. The vitreous hemorrhage in the right eye had partially regressed. The retina was attached but had a diffuse pale appearance due to retinal arterial occlusion, and there were widespread intraretinal hemorrhages. The optic nerve head was apparently absent (Figure 2).

**Figure 3:**
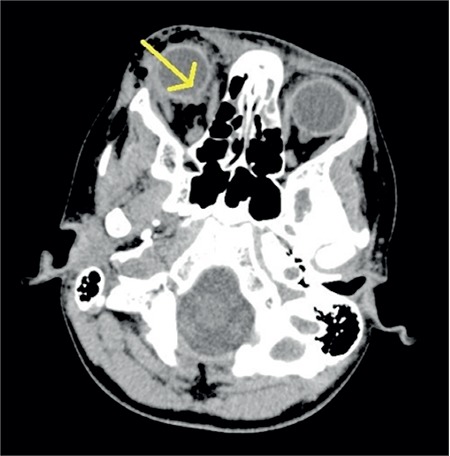
Computed tomography image taken one day after the trauma showing discontinuity of the right optic nerve in the sclera at the point of entry to the globe

Brain tomography conducted in the emergency department and orbital magnetic resonance imaging (MRI) examination requested by ophthalmology showed no pathology other than sporadic hemorrhages in the vitreous and irregularity at the lamina cribrosa level consistent with right optic nerve avulsion. Based on the results of ophthalmologic examination and imaging, the patient was diagnosed with optic nerve avulsion (Figure 3). Systemic steroid therapy was not expected to be of benefit to the patient and was discontinued.

Examination one week later revealed total retinal detachment in the right eye, which was attributed the trauma. No interventions were considered due to the lack of light perception in the right eye and the patient was scheduled for follow-up, but he did not return.

## Discussion

Avulsion of the optic nerve is a rare traumatic optic neuropathy which is currently untreatable, has poor visual prognosis, and occurs via indirect mechanisms and therefore, differentiating it from other traumatic optic neuropathies is important in terms of preventing unnecessary treatments and informing the patient.

Optic nerve avulsion can develop following blunt or penetrating ocular trauma through various indirect mechanisms. The optic nerve may become detached at the lamina cribrosa, which is the weakest point, due to trauma-related sudden increase in intraocular pressure, sudden posterior displacement of the optic nerve, and strong rotation or anterior displacement of the globe.^3^ While it often occurs as a result of blunt trauma to the eye or impact to the face,^4^ it occured after a penetrating orbital trauma in the present case. The only similar cases in the literature were described by Chaudhry et al.^5^ in a retrospectively analysis of 14 children with severe vision loss due to door-handle injuries. They noted that in all cases the pointed door handle had penetrated the orbit medial to the globe and suggested that this caused optic nerve avulsion by creating a wedge effect which pushed the globe against the lateral orbital wall and displaced it anteriorly. Similar mechanisms occur in both blunt and penetrating orbital traumas. The wedge effect causes sudden anterior movement of the globe and the optic nerve can separate from the lamina cribrosa.

Because optic nerve avulsion is usually accompanied by vitreous hemorrhage, diagnosis by ophthalmoscopic examination is not always possible in the acute phase. Especially in cases of partial avulsion, imaging methods may not show definitive signs and the diagnosis may be overlooked.^6^ Ocular ultrasound cannot be used in early evaluation of many patients due to the recent severe penetrating or blunt trauma.^7^ However, radiologic imaging methods should be utilized without delay if ophthalmoscopic examination is inadequate to establish a diagnosis in suspected cases of optic nerve avulsion.

Based on previous reports in the literature, retinal detachment is uncommon after optic nerve avulsion. Only a few case reports have described late fibrosis and subsequent tractional retinal detachment in some patients with long-term follow-up.^3^,^8^ In a case report by Mackiewicz et al.^9^ a patient who was treated with high-dose systemic steroids for a week underwent ultrasonography due to lack of visual improvement (no light perception) and persistent vitreous hemorrhage, and retinal detachment was observed. Vitrectomy was performed at two months, after which cystic gliosis and optic nerve avulsion were detected. There was no change in visual acuity after six months of follow-up. Likewise, retinal arterial occlusion is rarely associated with optic nerve avulsion.^10^ Our patient was distinct from other cases of optic nerve avulsion in that he exhibited both central retinal artery occlusion and early retinal detachment. The patient presented with a laceration in the superonasal region of the eyelid and was found to have complete avulsion of the optic nerve.

Although optic nerve avulsion can be diagnosed easily by ophthalmoscopic examination, accompanying vitreous hemorrhage can lead to delayed diagnosis and unnecessary high-dose systemic steroid treatment. Though rare, optic nerve avulsion should be considered in cases of severe post-traumatic vision loss when definite diagnosis cannot be established by ophthalmoscopic examination. Imaging with methods such as MRI and CT should be done promptly to diagnose pathologies of the globe and other intraorbital structures, including optic nerve avulsion.
